# Aptamers and Glioblastoma: Their Potential Use for Imaging and Therapeutic Applications

**DOI:** 10.3390/ijms18122576

**Published:** 2017-11-30

**Authors:** Emma M. Hays, Wei Duan, Sarah Shigdar

**Affiliations:** Centre for Molecular and Medical Research, School of Medicine, Deakin University, 75 Pigdons Road, Waurn Ponds, Victoria 3216, Australia; ehays@deakin.edu.au (E.M.H.); wei.duan@deakin.edu.au (W.D.)

**Keywords:** glioblastoma, aptamers, SELEX, cancer, targeted therapies, imaging, diagnosis, biomarkers, blood brain barrier

## Abstract

Glioblastoma is a highly aggressive primary brain tumour, renowned for its infiltrative growth and varied genetic profiles. The current treatment options are insufficient, and their off-target effects greatly reduce patient quality of life. The major challenge in improving glioblastoma diagnosis and treatment involves the development of a targeted imaging and drug delivery platform, capable of circumventing the blood brain barrier and specifically targeting glioblastoma tumours. The unique properties of aptamers demonstrate their capability of bridging the gap to the development of successful diagnosis and treatment options, where antibodies have previously failed. Aptamers possess many characteristics that make them an ideal novel imaging and therapeutic agent for the treatment of glioblastoma and other brain malignancies, and are likely to provide patients with a better standard of care and improved quality of life. Their target sensitivity, selective nature, ease of modification and low immunogenicity make them an ideal drug-delivery platform. This review article summarises the aptamers previously generated against glioblastoma cells or its identified biomarkers, and their potential application in diagnosis and therapeutic targeting of glioblastoma tumours.

## 1. Introduction

**Figure 1:**
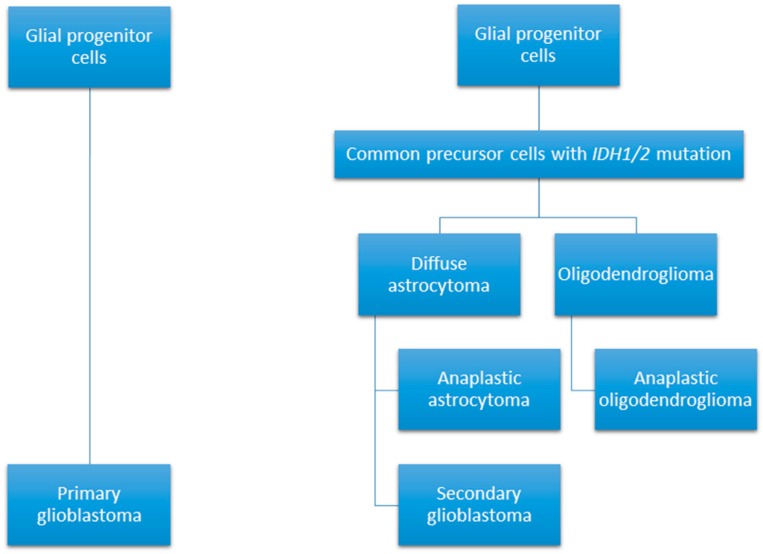
Glioblastomas can develop through two different pathways; arising as a primary malignancy; or through the progression of a lower grade glioma [11].

Glioblastoma or grade IV glioma is a highly malignant primary brain tumour with a particularly poor median survival duration of 14 months [1]. Whilst the incidence rate of glioblastoma currently stands markedly low at 3.2 per 100,000 population, its five-year survival rate is below 6% [2]. These tumours can arise spontaneously from glial cells, or can develop from the progression of a lower grade glioma and are defined as primary or secondary glioblastomas, respectively (see Figure 1) [3]. Histological features of glioblastoma include increased cellularity and angiogenesis, vascular proliferation, hemorrhage, necrosis, and cystic regions throughout the tumours [4,5]. Cell populations within these tumours tend to vary greatly, with the presence of both small, undifferentiated cells and large cells with multiple nuclei reported [4].

According to the World Health Organization, glioblastomas are grouped into three categories based on isocitrase dehydrogenase (IDH) status: glioblastoma IDH-wildtype, which is designated as primary or to have originated de novo; glioblastoma IDH-mutant, which is identified as a secondary tumour, arising from a lower grade glioma; and glioblastoma not otherwise specified (NOS) for tumours when IDH evaluation cannot be performed [6]. An estimated 90% of glioblastoma arise de novo, with the remaining 10% developing from a lower grade glioma [7]. Mutations of IDH lead to hypermethylation of histones and DNA, altering gene expression, promoting the activation of oncogenes and blocking tumour suppressing mechanisms [8]. A variety of genetic mutations are implicated in glioblastoma development including the *epidermal growth factor receptor* (*EGFR*), *human epidermal growth factor receptor two* (*ERBB2*), *isocitrase dehydrogenase one* (*IDH1*), *neurofibromin one* (*NF1*), *phosphoinositide three-kinase* (*PI3K*), *phosphatase and tensin homolog* (*PTEN*), *retinoblastoma protein* (*RB1*) and *tumour suppressor p53* (*TP53*) [9]. The gene expression patterns can be used to further categorise glioblastomas into four sub-categories: proneural; neural; mesenchymal; and classical [10].

The classical subtype is associated with amplification of chromosome 7 teamed with loss of chromosome 10, EGFRoverexpression and mutations. Mesenchymal glioblastomas maintain a high expression of *CH13L1*, *MET*, and genes associated with tumour necrosis factor and nuclear factor-κB pathways, along with mutations and deletions of NF1. The proneural subclass parallels secondary glioblastoma and lower-grade glioma with mutations in *IDH1* and *TP53*, and modification of platelet-derived growth factor receptor A (PDGFR-A) [10]. Finally, neural glioblastomas are similar to normal brain tissue; however, they do overexpress EGFR [10,12].The current treatment modality includes surgery, radiotherapy, and chemotherapy with the DNA-alkylating agent temozolomide [1,13]. Surgery is performed to debulk the tumour thereby reducing mass effect symptoms in the patient, while also allowing for the collection of tissue specimens for histologic analysis [14]. Glioblastomas are renowned for their heterogeneity and infiltrative growth; complete surgical resection is near impossible as a result, and further complicated by the inability to differentiate the tumour from normal brain tissue [15]. Radiation and chemotherapy form the next line of treatment, aiming to destroy the cancer cells that were missed or could not be removed during surgery [1]. Temozolomide is a DNA-alkylating agent capable of inducing single- and double-stranded breaks in DNA, resulting in senescence and cell death [16]. Whilst surgery and the addition of temozolomide during and post-radiotherapy has led to an increase in patient survival times, they are responsible for various adverse effects and poor quality of life in patients [13,17]. In addition, chemo-resistance and glioblastoma recurrence are inevitable for the majority of patients, highlighting the necessity of improved treatment options [16,18]. One of the greatest challenges facing modern medicine is the development of tumour targeting molecules, capable of specifically binding to their target with no adverse effects to normal cells and tissues within the body. Whilst the discovery and subsequent development of antibodies have become an integral part of scientific research, disease diagnosis and therapies, these molecules possess undesired characteristics and many pose significant risks to patients, limiting their clinical efficacy [19,20]. Antibody generation occurs in vivo, in both a time consuming and costly process, with incidences of great batch-to-batch variability [21]. The high cost of the complicated antibody production process limits their clinical use, particularly as large doses are needed for effective responses in patients [22]. Their large size hinders their ability to reach the desired targets due to poor tissue penetration, and can be irreversibly denatured by small changes in temperature, thereby limiting their shelf life and transport options [23]. Attempts have been made to humanize antibodies; however, complement-dependent toxicity (CDC), antibody-dependent cellular cytotoxicity (ADCC), and cytokine storms still occur in patients [24,25,26]. Despite these known issues, antibodies remain in development and clinical trials, while the development of alternatives with improved safety profiles are being investigated [27,28]. Targeted peptides and aptamers may bridge the gaps of diagnostic and therapeutic applications that are currently unfilled by antibodies; however, targeted peptides have been reviewed extensively elsewhere (see [29,30,31]) and fall beyond the scope of this review article. Targeted therapies will pave the way for personalized cancer medicine, ensuring patients receive treatment based on their tumour’s gene expression profiles, for effective treatment against glioblastoma and other currently incurable conditions.

Further understanding of the altered and uncontrolled signaling pathways in glioblastoma has potential to aid in the development of targeted treatments against the disease. Gene amplification and overexpression of the EGFR protein and its mutant variant EGFRvIII contributes to glioblastoma tumorigenesis and has been the target for new therapeutics [32,33]. Tyrosine kinase inhibitors of EGFR block downstream signaling pathways by inhibiting ATP binding to the intracellular domain, thereby impeding with responses leading to cell growth, invasion, and angiogenesis [34]. The use of the EGFR tyrosine kinase inhibitors erlotinib and gefitinib have been evaluated in numerous clinical trials, alone and in combination with standard glioblastoma treatment, with minimal effect on patient survival [35,36,37,38]. Monoclonal antibodies have also been investigated for the treatment of glioblastoma. In particular, bevacizumab was generated to inhibit vascular endothelial growth factor (VEGF) in order to prevent angiogenesis, survival and migration of glioblastoma tumour cells [39]. Two large phase III trials determined that the use of bevacizumab did not lead to increased survival compared to standard treatment, despite this, it has remained in use to treat recurrent glioblastoma when temozolomide rechallenge fails [40,41,42]. The unsuccessful development and clinical translation of more effective treatments for glioblastoma are hindered by the restrictive nature of the blood brain barrier.

The blood brain barrier’s role is to control the passage of molecules and cells into the brain, in order to protect this vital organ [43]. Tight junctions formed by a uniform monolayer of endothelial cells maintain an almost impermeable barrier, increasing the difficulty of conveying chemotherapeutics into the brain [44]. Great efforts have been made to develop mechanisms capable of delivering therapeutic agents across this barrier, including both invasive and non-invasive strategies. The proposed invasive strategies include temporary disruption of the blood brain barrier and intrathecal drug delivery [45,46,47,48]. A temporary disruption of the barrier can be achieved using focused ultrasound and its interaction with microbubbles to alter the structure of the endothelial layer [49]. However, this technique can lead to necrosis in the target tissue area, vasculitis, and seizures [50]. Intrathecal drug delivery introduces therapeutics via direct injection into the cerebrospinal fluid [48]. While this is a simple method of bypassing the blood brain barrier, the drugs are often filtered from the cerebrospinal fluid before they reach the targeted area, and the use of a catheter often results in infection [47,51]. A less invasive method involves chemically altering drugs, potentially by the addition of a lipid-like structure, or modifying the drug to suit specific receptors, where it would be converted within the target organ to the active state [52]. Modifying drugs may result in considerable changes from the parental drug, in turn altering the pharmacodynamics and biological efficacy [44]. Finally, biological delivery systems can be used to increase the uptake of drugs by transporters that would normally be used for essential nutrients [53]. 

**Figure 2:**
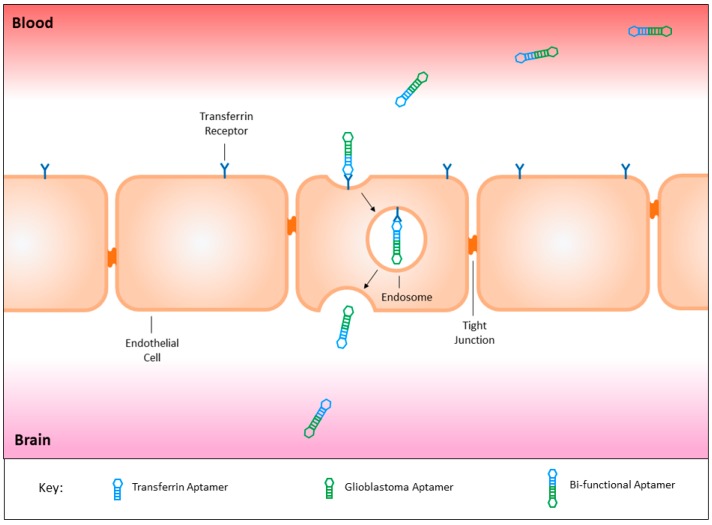
The fusion of a transferrin aptamer with a glioblastoma targeting aptamer would create a bi-functional aptamer capable of crossing the blood brain barrier to target glioblastoma tumour tissues specifically. This targeted mechanism may be utilised for the safe delivery of both imaging and therapeutic agents, sparing healthy cells throughout the body and the surrounding brain tissues from off-target effects.

The presence of transport molecules on the surface of the blood brain barrier have recently proven to be an effective mechanism to utilise for the delivery of substances into the brain via receptor-mediated endocytosis. Yu and colleagues successfully utilised the transferrin receptor to transport an antibody across the endothelial layer into the brain itself [54]. On the contrary, the use of antibodies is associated with an immune response, therefore the development of a novel targeting agent similar to antibodies is required for the safe treatment of brain malignancies [55]. The recent development of a bi-functional aptamer capable of transcytosing across the blood brain barrier aided by the transferrin receptor in vivo, can be conjugated to a glioblastoma targeting aptamer (Figure 2), and potentially improve its treatment modality [56].

## 2. Aptamers

**Figure 3:**
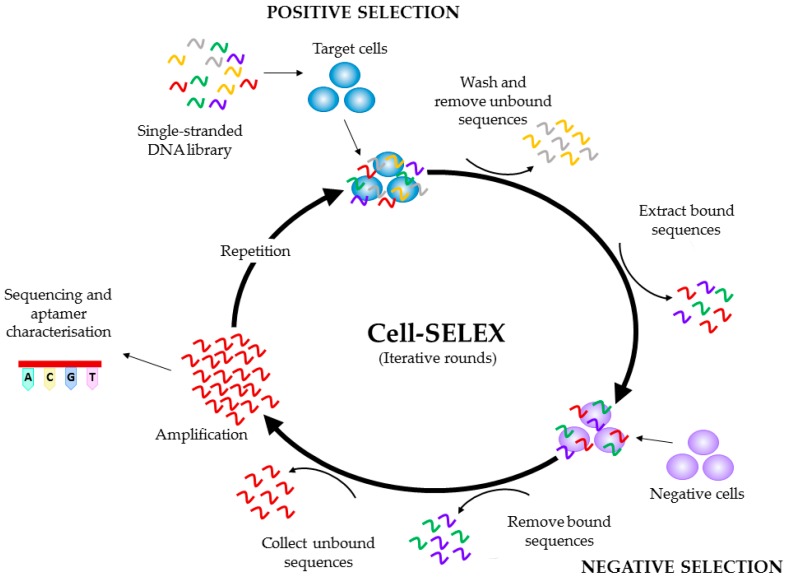
Schematic representation of a Cell-SELEX (systematic evolution of ligands by exponential enrichment) protocol. Initially, a single-stranded DNA library is incubated with the target cells. The cells are washed to remove the unbound sequences, and the bound sequences are collected, prior to incubation with negative (control) cells. The bound sequences are removed and discarded, whilst the unbound sequences are amplified and used to begin the next round of SELEX. This cycle is repeated a number of times, before the pool is sequenced and characterised. The bold arrows denote one iterative round of SELEX, with the small arrows depicting the different steps of the protocol: incubation of the single-stranded DNA library with the target cells; washing and removal of the unbound sequences, incubation with negative cells; removal of bound sequences; collection of bound sequences; amplification and cycle repetition prior to sequencing and aptamer characterization.

Aptamers, also referred to as ‘chemical antibodies’ are single stranded oligonucleotides capable of binding to a target via shape recognition, in a similar fashion to antibodies [57]. Aptamers are generated against specific targets via the process of the systematic evolution of ligands by exponential enrichment (SELEX) and their sequence can be modified to optimise binding affinity and selectivity (see Figure 3) [58,59,60,61]. The selection of aptamers occurs in vitro, as does their subsequent manufacture, leading to little to no variation between batches, low immunogenicity, and are a considerably smaller size than antibodies, further increasing their ease of tissue penetration [62]. The enhanced binding properties of aptamers can saturate the available binding sites on the tumour surface, and therefore lead to improved imaging signals and intratumoural delivery of therapeutic agents, comparative to antibodies [22,63]. Chemotherapeutic agents can be attached to aptamers in order to deliver them to tumour cells, thereby reducing unwanted off-target effects, a particularly important factor for the development of new therapeutic agents for brain tumours [57]. Specificity of aptamer binding highlights their suitability for tumour imaging and targeted drug delivery, particularly in an organ as important as the brain. The attachment of imaging agents to aptamers has previously been achieved, and may lead to the development of more effective tumour imaging strategies for both histologic analysis of tissues as well as determining tumour location within the patient [64,65,66,67]. The use of aptamers for both imaging and therapeutic delivery have been reviewed extensively elsewhere [68,69,70,71].

## 3. Glioblastoma Targeting Aptamers

**Figure 4:**
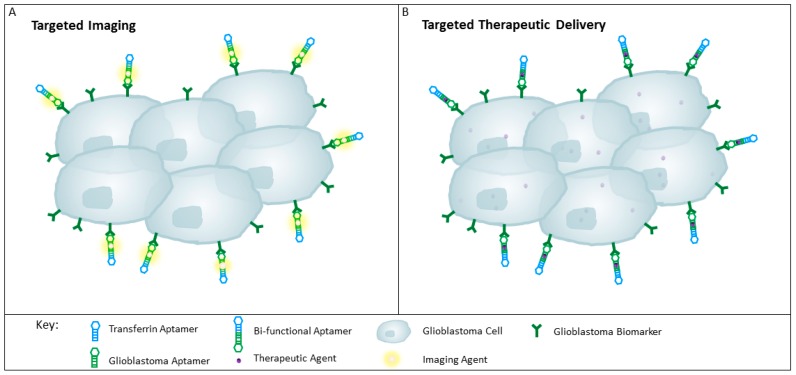
Schematic representation of aptamers for targeting glioblastoma tissue. (**A**) Targeted delivery of imaging agents to glioblastomas with the use of a bi-functional aptamer conjugated with an imaging agent such as radionuclides, may improve tumour detection; (**B**) Therapeutic agents can be conjugated to the bi-functional aptamer for the specific delivery to the glioblastoma cells, thereby reducing unwanted off-target effects and sparing the healthy surrounding tissues and throughout the rest of the body.

To date, a multitude of aptamers have been generated against cell membrane proteins expressed on glioblastoma cells (see Table 1 and Table 2). These aptamers have potential to be used to identify new biomarkers, and to specifically deliver imaging or therapeutic agents to revolutionise glioblastoma diagnosis and treatment (see Figure 4). 

### 3.1. Aptamers Targeting Tumour Intitiating Cells

A relatively new concept in cancer biology is the discovery of tumour initiating cells (TIC), referred to as cancer stem cells, thought to be a driving force in tumour development and therapeutic resistance [94]. While a population of TIC in glioblastomas can be identified by the glycoprotein CD133, some CD133-negative cells in these tumours have the ability to self-renew, thus this marker may not be indicative of all stem cells within glioblastoma tumours [95]. It should be noted that the CD133 receptor, usually detected with the AC133 epitope monoclonal antibody, can become truncated, thereby hindering the antibody’s access to its binding region and leading to false-negative CD133 expression results in cancer cell populations [96]. As such, CD133 expression should be confirmed with multiple techniques. Despite these controversies, CD133 is still an attractive therapeutic target that requires further investigation due to its presence within these tumours. 

To develop aptamers against CD133, Shigdar et al. utilised differential cell-SELEX with HEK293T-CD133 transfected cells and normal HEK293T cells as a negative control, to ensure the aptamers were binding to the desired epitope [90]. The generation of RNA containing 2′-F-pyrimidine aptamers capable of binding to CD133 was confirmed using both CD133 positive, and negative cell lines, via flow cytometry and confocal microscopy. The CD133-A15 aptamer binds to the AC133 epitope, similar to that of the standard antibody used for this receptor’s detection. In contrast, CD133-B19 does not bind to the AC133 epitope and therefore has potential to be used to detect cancer stem cells within glioblastoma tumours, as well as potentially deliver therapeutics to these target cells. 

Differential cell-SELEX was also utilised to generate the aptamers A3 and A4 against glioblastoma TIC, by using cells extracted from human glioblastoma tissue xenografts [72]. The collected cells were sorted based on CD133 expression and used for positive and negative selection in all SELEX rounds, with the addition of human neural progenitor cells as an extra counter selection step to promote binding to a cancer surface marker. This selection process developed aptamers capable of specific binding and uptake to glioblastoma TIC, generating a potential method for their detection and treatment. Further characterisation of the identified aptamers is required to determine their molecular targets and determine their efficacy as diagnostic or therapeutic delivery agents. 

### 3.2. Aptamers Targeting Tenascin-C

Tenascin-C is a large glycoprotein found in the extracellular matrix [73]. While normal adult tissues have little or no expression, higher levels are present during foetal development, wound healing, atherosclerosis, psoriasis, and tumour growth [97,98]. The high levels of tenascin-C expression in tumours is associated with angiogenesis, and may be an effective biomarker for diagnosis and treatment [73]. 

The aptamer TTA1 was developed via a crossover-SELEX protocol with U251 glioblastoma cells and purified human tenascin-C [73]. This aptamer was generated with RNA containing 2′-F-pyrimidine to induce resistance to nucleases in the blood, and was truncated prior to further modifications to improve aptamer stability and half-life with the substitution of 2′-OCH_3_ purines, the addition of a thymidine cap at the 3′ end, and a 5′ amine as a conjugation site. In vitro testing determined the aptamer to bind to human tenascin-C with high affinity, with a 20-fold reduction in affinity to the mouse protein. The addition of the conjugate site on TTA1 enables the attachment of various molecules, ensuring its adaptability for various clinical applications. ^99m^Tc radiolabelled-TTA1 administered intravenously to nude mice successfully targeted tumour xenografts of U251 glioblastoma cells and MDA-MB-435 breast cancer cells, and its rapid clearance from the blood and uptake into the tumours indicates TTA1 as a viable tumour imaging modality [66]. It should be noted that there are limitations with the use of animals as human disease models, as xenografts do not wholly replicate the disease that occurs in humans [99]. The use of cultured cells in xenografts can lead to tumours with genetic profiles differing greatly from patient tumours and therefore do not always accurately represent treatment outcomes in patients. The use of xenograft models for glioblastoma tumours completely fails to replicate the cancer’s microenvironment, and indeed the ability of these aptamers to cross the blood brain barrier, as these xenografts are heterotopic. Despite these drawbacks, animal models still provide important information for translational drug development prior to experimental clinical trials, and give great insight into efficacy of newly developed imaging agents. It is vital that the TTA1 aptamer is tested in healthy animal models to determine if it is capable of specifically targeting tumour tissue and also asses its ability to cross the blood brain barrier. If TTA1 is not able to do so, this aptamer may be attached to a transferrin aptamer to ensure its successful transition into the brain. TTA1 must also be tested in animal models that provide a relevant clinical representation of glioblastoma, to ensure its effectiveness as both a diagnostic and therapeutic delivery agent.

Another aptamer capable of specifically binding to tenascin-C is GBI-10, a DNA aptamer generated against U251 glioblastoma cells. Radiolabeling GBI-10 with positron emission tomography (PET) isotopes, ^18^F and ^64^Cu, developed aptamers capable of imaging tenascin-C within U87MG glioblastoma and MDA-MB-435 breast cancer xenografts in mice [74]. The aptamer’s rapid clearance hindered intratumoural uptake; although these properties are ideal for PET imaging agents, they would need to be modified for effective therapeutic delivery in in vivo systems. To improve GBI-10’s stability and affinity, d-/l-isonucleotides and 2′-dI phosphoramidite were incorporated into the aptamer structure, and successfully increased the binding affinity to U251 cells in vitro [75]. Aptamer binding affinity and serum stability are vital characteristics that determine their efficacy as diagnostic or drug-delivery platforms. Modifications to aptamers to improve their half-life and targeting ability may be vital to ensure their success in clinical applications. Further testing is warranted in more appropriate glioblastoma animal models to prove GBI-10’s capability to specifically target glioblastoma tissues in a clinically relevant in vivo system. 

### 3.3. Aptamers Targeting Platelet Derived Growth Factor Receptor β

Overexpression and mutations of the platelet derived growth factor receptor (PDGFR) are associated with gliomagenesis and tumour progression [100]. In glioblastoma, deregulation of the PDGFR signaling cascade and overexpression of this receptor is frequently displayed, indicating it as a potential therapeutic target [101].

Cell-SELEX was employed to develop a 2′-F-pyrimidine containing RNA aptamer capable of recognising platelet derived growth factor receptor β (PDGFRβ) [76]. This aptamer, Gint4.T, was determined to internalise into the U87MG glioblastoma cells, indicating its ability to be developed as a targeted delivery platform of therapeutics to the tumour tissues. Binding of this aptamer to the PDGFRβ was found to interfere with the receptor’s signaling cascade by preventing phosphorylation of the tyrosine kinase domains, thereby reducing cell proliferation and migration both in vitro and in athymic CD-1 nude mice tumour xenografts in vivo. To prove Gint4.T’s success as a therapeutic delivery platform, this aptamer was conjugated with anti-microRNA-10b to counteract expression of the oncogenic microRNA-10b seen in glioblastoma cancer stem cells, and induced cellular differentiation, thereby preventing tumoursphere growth and development when combined with GL21.T conjugated with microRNA-37 [77]. Interestingly, Gint4.T was capable of crossing the blood brain barrier in a physiologically relevant in vitro model previously developed by Kumar and associates (see [102]), likely via receptor mediated transcytosis, although this is yet to be replicated in an in vivo model. The ability of this aptamer to cross the blood brain barrier and specifically target glioblastoma cancer stem cells may lead to an effective therapeutic against this normally evasive cell population, thereby improving prognosis and increasing survival times for glioblastoma patients. 

### 3.4. Aptamers Targeting Axl

Axl is a receptor tyrosine kinase commonly overexpressed in glioblastomas and other systemic tumours, associated with cancer cell survival, angiogenesis, proliferation, invasion, and motility [103,104,105]. The GL21.T aptamer is capable of binding and inhibiting Axl signaling, and may serve as a novel therapeutic agent to control tumour cell growth and invasion driven by this oncogenic protein [78]. This aptamer reduced migration and invasion of A549 lung cancer cells and U87MG glioblastoma cells in vitro and in athymic CD-1 nude mice xenografts in vivo. As previously mentioned, GL21.T conjugated with microRNA-37 used in combination with microRNA-10b-conjugated Gint4.T, transformed tumourspheres into adherent-like cells, inhibiting their ability to grow in spheroid form [77]. GL21.T was also able to traverse the in vitro blood brain barrier model, if this aptamer is capable of crossing this barrier in vivo, it could potentially be developed as a therapeutic targeting agent in the clinical setting. However, previous attempts to inhibit PDGFR in clinical trials have failed due to tumour resistance and pathway compensatory mechanisms [79,80]. Therefore, GL21.T should be used as a therapeutic delivery platform, and used in combination with other targeted therapies to ensure complete tumour eradication. 

### 3.5. Aptamers Targeting the Epidermal Growth Factor Receptor

EGFR is involved with important cellular functions including cell growth, differentiation, survival, and migration, and deregulation of its signaling cascade is a driving force of glioblastoma tumorigenesis [32,106]. An aptamer capable of targeting EGFR and the mutant variant EGFRvIII would serve to be an ideal candidate for use in glioblastoma therapy, as their overexpression and mutation are prevalent in the majority of glioblastoma tumours [32]. 

An anti-EGFR RNA aptamer, CL4, capable of binding to both the wildtype and mutant variant has previously been generated via differential cell-SELEX with lung cancer cell lines [91]. Upon binding to the target receptor, CL4 prevented tyrosine-phosphorylation in a time-dependent manner, decreasing cell viability, proliferation, migration, and invasion in vitro [91,92]. Whilst minimal in vivo work has been undertaken with CL4, promising results from a triple-negative breast cancer xenograft model in athymic CD-1 nude mice determined this aptamer was capable of reducing tumour size, without inducing toxicity in the treated mice [93]. CL4 displays a therapeutic effect against EGFR activation; however, EGFR inhibitors have failed to prove success as monotherapies for glioblastoma, as such, CL4 should be used as a shuttle for therapeutic or diagnostic agents to these tumours in order to have clinical success [107].

Differential cell-SELEX developed an aptamer, U2, with U87MG cells expressing EGFRvIII or wildtype EGFR as positive and negative selectors, respectively [81]. This aptamer’s high affinity for the membrane receptors indicated its potential use as a diagnostic agent. Therefore, U2 was radiolabelled with ^188^Re to investigate its ability to function as a molecular probe in EGFRvIII expressing glioblastoma xenografts in male BALB/c nude mice. This aptamer successfully accumulated in the target tumours, highlighting its potential to be used as an effective glioblastoma imaging tool. There is little evidence to date that suggests U2 can penetrate the blood brain barrier in these mice, as such, conjugation with a transferrin receptor aptamer would ensure effective transport into the brain, though this would need to be extensively investigated both in vitro and in vivo. Tan et al. created aptamer 32 via differential cell-SELEX, utilising U87MG-EGFRvIII cells as the target for aptamer selection and U87MG cells as a negative control [82]. Binding assays were performed to evaluate aptamer specificity, with aptamer 32 showing binding to the U87MG-EGFRvIII target cells, and no binding to U87MG cells or the kidney cell line HEK293. Pull-down assays and confocal microscopy concluded the aptamer’s target was EGFRvIII, and the aptamer was translocated to the nucleus of target cells, indicating its potential to be used for the targeted delivery of therapeutics within glioblastoma tumours. A follow-on study conjugated c-Met small interfering RNA (siRNA) to aptamer 32, and successfully delivered the siRNA to target U87MG-EGFRvIII cells, inhibiting the target gene’s expression and cell proliferation whilst inducing apoptosis [83]. Conjugation of aptamer 32 to quantum dots could verify the expression of EGFRvIII in various glioblastoma tissues, the presence of this receptor was confirmed with immunohistochemistry [84]. This novel imaging probe was also capable of crossing the blood brain barrier in vivo, and accumulated in tumour tissues within the brains of male nude mice. Analysis of mouse body weight and major organs determined that the quantum dot-aptamer conjugates did not induce toxicity in the animals, further highlighting this aptamer’s potential for use in the clinical setting. Overall, these results indicate aptamer 32 as a candidate tumour imaging probe and as a therapeutic delivery platform for the treatment of glioblastoma, though further investigation is required to determine aptamer 32’s mechanism of crossing the blood brain barrier, and to further determine its stability and specificity to glioblastoma tumours within the brain.

An alternative SELEX method was employed to develop a 2′-F-pyrimidine containing RNA aptamer against human EGFR protein [85]. This aptamer, E07, was determined to competitively bind to both the wildtype and mutant variant three of EGFR, hindering ligand binding and autophosphorylation of these receptors in vitro. E07 has since been utilised to successfully capture and isolate circulating tumour cells with the use of biochips in vitro, with potential to be used for cancer diagnosis and evaluation of metastasis in patients [86,87]. 

### 3.6. Targeting Nucleolin with a Guanine-Rich Oligonucleotide

Nucleolin is associated with ribosome biogenesis, and the overexpression of this protein in cancer cells is linked to cell division, angiogenesis and inhibiting apoptosis, and therefore, is a viable target for delivery of therapeutic agents to glioblastomas, or other forms of cancer [108,109]. The development and use of guanine-rich oligonucleotides (GRO) stems from their intriguing biological properties, notably, their enhanced uptake that could aid in the delivery of therapeutic agents to target tissues, although they are known to display non-specific effects [110,111]. AS1411 is a GRO, utilised for its antiproliferative effects and nucleolin targeting ability in vitro [112]. The efficacy of AS1411 as a cancer therapy was replicated in vivo, as intraperitoneal injections were able to significantly reduce pancreatic cancer xenograft growth in mice. In terms of using AS1411 as a treatment for glioblastoma, Luo et al. successfully delivered AS1411-conjugated paclitaxel nanoconjugates to U87MG-PMT48 orthotopic glioblastoma xenografts in BALB/c nude mice, significantly increasing the median survival time for the treated mice [113]. Despite these promising results, the clinical translation of AS1411 was not so successful, with evaluation as a therapeutic in a phase II trial for metastatic renal cell carcinoma, only one patient out of 35 responded to the treatment [114]. Rigorous evaluation of AS1411 as a glioblastoma treatment in physiologically relevant in vitro and in vivo models is vital to ensure successful transition into clinical trials, and the potential off-target uptake must be evaluated if AS1411 is to be used for targeted drug delivery in the future. 

### 3.7. Glioblastoma Aptamers with Unknown Targets

Cell-SELEX can be advantageous for aptamer development as whole cells have a myriad of surface markers that aptamers could bind to, many which remain unidentified. Therefore, aptamers can be generated against unknown surface markers that can later be identified, improving our knowledge of glioblastoma and other cancers. 

Cell-SELEX was employed to develop DNA aptamers using the A172 glioblastoma cell line [88]. The sequenced aptamers GMT 3, GMT 4, GMT 5, GMT 8, and GMT 9 bound to glioblastoma cells with high affinity, and were further characterised to determine if binding was specific to glioblastoma cell lines. GMT 4 and 8 bound to the CEM leukemia cells and breast cancer cells GMBJ1, indicating that these cells share a surface marker recognised by these aptamers. Whilst these aptamers did bind to the glioblastoma cells, they had increased affinities to one of each leukemic and breast cancer cell line by more than two-fold. As such, these aptamers may have potential for use as a targeted imaging or therapeutic delivery strategy in these cancers as well. Treatment with proteinase K and trypsin was performed to determine if the aptamer binding targets were membrane proteins. All aptamers lost binding affinity after proteinase K treatment, indicating all of the aptamers likely bind to proteins. Trypsin application lead to a slight decrease in GMT 3 and 5 binding, demonstrating their targets are resistant to trypsin cleavage. These results show that negative selection is vital to cell-SELEX to identify aptamers specific to a tumour type, and to date, no publications have indicated the targets of these aptamers. 

Differential cell-SELEX generated aptamers GBM128 and 131 using U118-MG cells for positive selection and astroglial cells SVGp12 for negative selection [89]. These aptamers were found to bind to the U118-MG glioblastoma cells in vitro, and did not recognise the astroglial cell line used in the SELEX process. Further testing with paraffin embedded tissues determined these aptamer’s ability to bind to glioblastoma and glioma tissues, with no binding observed in the astroglial tissues. Whilst these aptamers could not differentiate between glioblastoma and other glioma tumours, they did not bind to normal brain tissue, indicating their potential use in glioma diagnosis. Further investigation is required to determine the aptamers’ targets and prove their efficacy for glioma diagnosis. 

### 3.8. Circumventing the Blood Brain Barrier

Whilst there are a number of aptamers that have been generated against cancer cells or their surface markers, the majority have only been investigated in vitro. Although the results show good selectivity, specificity, and efficacy, the challenge will be to see if these aptamers can cross the blood brain barrier in clinical models. One way of rescuing these aptamers, should they fail, is to use a transferrin receptor aptamer for receptor-mediated transcytosis in order to give these aptamers access to the brain. This mechanism has been successfully utilised by Macdonald et al. with a bi-functional aptamer capable of transcytosing through an in vitro blood brain barrier model, and also confirmed to reach the brain of a healthy mouse model following intravenous injection [56]. The conjugation of aptamers seen in the previous paper serves as a proof of concept that combining a cancer-targeting aptamer to a transferrin receptor aptamer, can develop a bi-functional aptamer capable of transcytosing through the blood brain barrier, to specifically target tumours within the brain.

## 4. Conclusions

Brain cancers, particularly glioblastomas, have very poor five-year survival rates and there is an urgent need to develop new therapeutic strategies with superior mechanisms of action to the current available therapies. The issue of getting new therapies across the blood brain barrier has been investigated in a number of studies, and there are now several strategies available to actively transport therapeutics through the barrier in order to reach the cancer cells within the brain. Whilst some of these transport mechanisms are unlikely to provide the patient with a better standard of care and improved quality of life, there are some with potential to revolutionise the treatment of glioblastoma, and other brain disorders. 

Aptamers possess many characteristics that make them an ideal novel therapeutic agent for the treatment of glioblastoma and other brain malignancies. Their target sensitivity, selective nature, ease of modification and low immunogenicity make them an ideal drug-delivery platform. The development and optimisation of aptamers capable of transcytosing through the blood brain barrier to specifically deliver imaging and chemotherapeutic agents to glioblastoma tissues have potential to revolutionise brain tumour treatment. 

In conclusion, aptamers show great promise as novel agents for tumour detection and treatment, although ongoing investigations are required to ensure their effective clinical translation. The research performed to date indicates aptamers are an innovative diagnostic and therapeutic platform for glioblastomas. Furthermore, these novel nucleic acids are improving our understanding of cancer and glioblastoma biology. 

## Figures and Tables

**Table 1:** Glioblastoma aptamers and their SELEX methods.

Aptamer	Target	SELEX Method	Positive Selection	Negative Selection	Reference(s)
**A3, A4**	Unknown	Differential cell-SELEX	CD133+ TIC	CD133− cells; human neural progenitor cells	[72]
**TTA1**	Tenascin-C	Crossover-SELEX	U251 cells; human Tenascin-C	No negative selection	[66,73]
**GBI-10**	Tenascin-C	Cell-SELEX	U251 cells	No negative selection	[74,75]
**Gint4.T**	PDGFRβ	Cell-SELEX	U87MG cells	No negative selection	[76,77]
**GL21.T**	Axl	Differential cell-SELEX	U87MG cells	T98G cells	[77,78,79,80]
**U2**	EGFRvIII	Differential cell-SELEX	U87MG-EGFRvIII cells	U87MG cells	[81]
**Aptamer 32**	EGFRvIII	Differential cell-SELEX	U87MG-EGFRvIII cells	U87MG cells	[82,83,84]
**E07**	EGFR; EGFRvIII	Protein-SELEX	Human EGFR	No negative selection	[85,86,87]
**GMT 3–9**	Unknown	Differential cell-SELEX	A172 cells	No negative selection	[88]
**GBM128, GBM131**	Unknown	Differential cell-SELEX	U118-MG cells	SVGp12 cells	[89]

**Table 2:** Aptamers generated against potential biomarkers for glioblastoma targeting.

Aptamer	Target	SELEX Method	Positive Selection	Negative Selection	Reference(s)
**CD133-A15, CD133-B19**	CD133	Differential cell-SELEX	HEK293T-CD133 cells	HEK293T cells	[90]
**CL4**	EGFR; EGFRvIII	Differential cell-SELEX	A549 cells	H460 cells	[91,92,93]

## Abbreviations

ADCC	Antibody-dependent cellular cytotoxicity
ATP	Adenosine triphosphate
CDC	Complement-dependent cytotoxicity
DNA	Deoxyribose nucleic acid
EGFR	Epidermal growth factor receptor
EGFRvIII	Epidermal growth factor receptor variant three
ERBB2	Human epidermal growth factor receptor two
NF1	Neurofibromin one
NOS	Not otherwise specified
PDGFRA	Platelet derived growth factor receptor A
PDGFRβ	Platelet derived growth factor receptor β
PI3K	Phosphoinositide three-kinase
PTEN	Phosphatase and tensin homolog
SELEX	Systematic evolution of ligands by exponential enrichment
PET	Positron emission tomography
RB1	Retinoblastoma protein
RNA	Ribonucleic acid
siRNA	Small interfering RNA
TIC	Tumour initiating cells
TP53	Tumour suppressor p53
VEGF	Vascular endothelial growth factor
